# Lean Management Approach for Reengineering the Hospital Cardiology Consultation Process: A Report from AORN “A. Cardarelli” of Naples

**DOI:** 10.3390/ijerph19084475

**Published:** 2022-04-08

**Authors:** Eduardo Bossone, Massimo Majolo, Serena D’Ambrosio, Eliana Raiola, Michele Sparano, Giuseppe Russo, Giuseppe Longo, Maria Triassi, Angelo Rosa

**Affiliations:** 1 A.O.R.N. “Antonio Cardarelli”, 80131 Naples, Italy; ebossone@hotmail.it (E.B.); massimo.majolo@aocardarelli.it (M.M.); eliana.raiola@aocardarelli.it (E.R.); michele.sparano@aocardarelli.it (M.S.); ariete_gr@liberto.it (G.R.); glongo@gmail.com (G.L.); 2 Department of Electrical Engineering and Information Technology, University of Study of Naples “Federico II”, 80125 Naples, Italy; 3 Department of Public Health, University of Naples “Federico II”, 80131 Naples, Italy; triassi@unina.it; 4 Interdepartmental Center for Research in Healthcare Management and Innovation in Healthcare (CIRMIS), University of Naples “Federico II”, 80131 Naples, Italy; 5 Department of Management, Finance and Technology, University LUM “Giuseppe De Gennaro” of Casamassima, 70010 Puglia, Italy; rosa@lbsc.it

**Keywords:** lean management, quality improvement, teleconsulting, process re-engineering

## Abstract

Background: Consultations with specialists are essential for safe and high-quality care for all patients. Cardiology consultations, due to a progressive increase in cardiology comorbidities, are becoming more common in hospitals prior to any type of treatment. The appropriateness and correctness of the request, the waiting time for delivery and the duration of the visit are just a few of the elements that can affect the quality of the process. Methods: In this work, a Lean approach and Telemedicine are used to optimize the cardiology consultancy process provided by the Cardiology Unit of “Antonio Cardarelli” Hospital of Naples (Italy), the largest hospital in the southern Italy. Results: The application of corrective actions, with the introduction of portable devices and telemedicine, led to a reduction in the percentage of waiting for counseling from 29.6% to 18.3% and an increase in the number of patients treated. Conclusions: The peculiarity of the study is to apply an innovative methodology such as Lean Thinking in optimizing the cardiology consultancy process, currently little studied in literature, with benefits for both patients and medical staff.

## 1. Introduction

Clinical consultation offers patients an important opportunity to participate in the healthcare process and interact with a range of healthcare professionals [1]. From this interaction, it is possible to provide the main means for a correct diagnosis, for the treatment of diseases and for the prevention of health problems [2,3,4,5].

The growing complexity of cardiology care is associated with the progressive aging of the population, more often affected by comorbidities [6,7]. In the hospital setting, a formal cardiology consultation is often used. The cardiologist evaluates the clinical situation and offers specific recommendations regarding diagnosis and treatment, recommending any further diagnostic tests or a specific drug therapy, in a written note that becomes a permanent and integral part of the medical record [5]. In accordance with guidelines on the subject [8,9], it is a key step in cardiologic risk assessment for noncardiac procedures [10,11,12,13].

To ensure the effectiveness, efficiency and appropriateness of all hospital processes, health managers have sought improvement strategies based on objective data in several areas. Several studies have shown that the support of biomedical data and signal analysis by means of advanced techniques has proved to be successful in clinical practice, with particular regards to the development of decision-making support systems based on artificial intelligence and statistical analysis [14,15,16,17,18,19,20,21]. In addition, the combined use of managerial approaches organization together with data analysis techniques showed to be a promising tool in the healthcare management. In particular, a paradigm increasingly used for the management of healthcare processes is the Lean Thinking [22,23,24].

Lean Thinking is based on the concept of continuous improvement focusing on flow, on the concept of value aiming at reducing waste. Although lean was born in an industrial context, it quickly spread to other contexts, reaching as far as healthcare. This concept applied in healthcare is called ‘lean healthcare’.

Thanks to the introduction of Information and Communications Technology (ICT), it was possible to achieve further significant improvements. Telemedicine is the use of telecommunications to provide remote health care [25]. The adoption of telemedicine has allowed better access to care, improved re-source efficiency and a reduction in the costs associated with traditional visits [26,27]. Specifically, electronic cardiovascular counseling is a new line of services in advisory medicine and represents a cost-effective and safe alternative to providing advisory cardiovascular care. [28] A further push in the diffusion of these solutions came due to the COVID-19 emergency, where teleconsultation was used as an effective tool for reducing the risk of transmission for both healthcare workers and physicians [10,29,30,31].

In this work, a Lean approach is used to optimize the cardiology consultancy pro-cess provided by the Cardiology Unit of “Antonio Cardarelli” Hospital of Naples (Italy), which provides consultancy services to 43 Units including COVID-19 positive patients. To do this, both alternative delivery methods, such as teleconsultation, and the use of portable biomedical technologies, such as ultrasound scans, widely discussed in the literature [32,33], will be introduced as improvement/corrective actions within the Lean Thinking approach.

## 2. Materials and Methods

**Figure 1:**
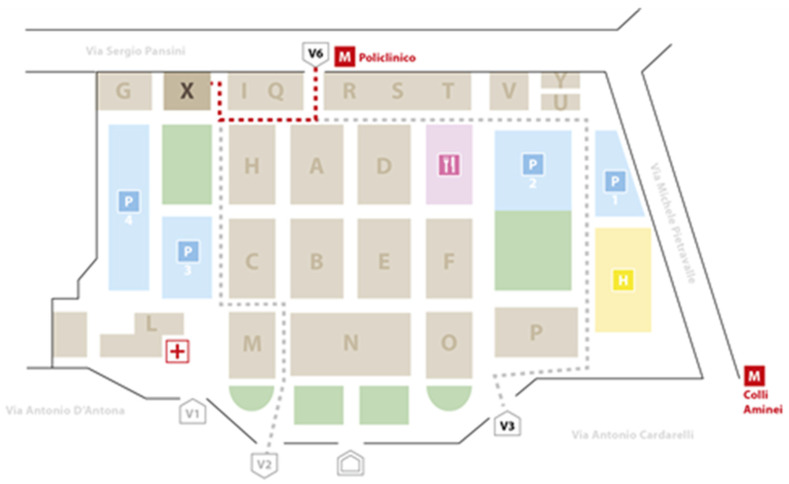
A.O.R.N. “A. Cardarelli” of Naples.

In this study, the main principles and tools of Lean Thinking have been applied in order to eliminate the wastes in the cardiology consultancy process (the so-called “muda” according to the Lean theory), i.e., by identifying, understanding and reduce the incidence of those process activities or steps that do not create value in the customer’s perception. The study was conducted at the A.O.R.N. “A. Cardarelli” Hospital (Naples, Italy), representing a center of national relevance and the largest hospital in the southern Italy. In fact, the “Antonio Cardarelli” National Importance Hospital is the first hospital in Campania (Italy), in terms of size and quality-quantity offer. The organizational structure of the Hospital identifies each single building (pavilion) with the letters of the alphabet, as shown below in Figure 1, occupying a total area of 250,000 square meter of these, 50,000 square meters are represented by buildings, and the remaining 200,000 by tree-lined avenues and pinewood:

The mission of the A.O.R.N. “Antonio Cardarelli” is to be an acute care hospital that provides highly effective and quality diagnosis, treatment, and rehabilitation services; it offers emergency (with over 300 daily accesses) and in election, in ordinary hospitalization, day-surgery, day-hospital and outpatient services. The hospital has a role of National Importance of particular importance for the offer of health services of excellence, for the demonstrated ability to design and implement innovations in the health area, for the role assumed in the sector of scientific and technical research, in the training of doctors and health professions. In the year 2021, the structure has 1060 beds in assets divided between the 99 different medical specialties present and related to eight health departments.

### 2.1. Principles of Lean Thinking

The application principles of the Lean approach followed in this study are the following:

1. Value: define the value of the process and the sources of waste.
2. Value stream: identify the value stream that describe the process.
3. Flow: make the value flow across the process steps.
4. Pull: make the flow pulled by the customers.
5. Perfection: continue to improve to pursue the perfection (i.e., the total absence of wastes).

These five principles represent the basic elements for an effective Lean project implementation. In this study, each of these principles has been adopted in distinct phases of the project and through different managerial tools, as described in the following paragraphs.

### 2.2. Goal Definition and Team Building

As a first step of the methodology, the general objective of the project was defined, and a qualified multidisciplinary team aimed towards the achievement of the specific and shared goal was formed. In particular, the general aim of the project was the improvement of the cardiology consultancy process by reducing the waiting time for the consultation, i.e., the difference between the time when the consultation is requested and the time when the consultation is carried out, which is termed as the Critical to Quality (CTQ) of the process, as summarized int the following Equation (1):

CTQ = Waiting Time for the consultation = WTc = Tserved − Trequested(1)

where Tserved and Trequested are the time of the consultation execution and the time of the consultation request, respectively.

To pursue this goal, the project management team was constituted by the following stakeholders: doctors, nurses, health managers, engineers and all other professionals included in the process.

### 2.3. Study of the as-is Process: Identify the Value and the Value Stream

The second phase of the Lean approach involves identifying the activities that make up the patient’s overall care path in the current state. For the purposes of the project, joint meetings were held between the management team member in order to examine the process under study and identify the critical aspects by classifying the process activities in “value-added” and “non-value-added” ones.

The fundamental Lean tool that was adopted in this phase is the Value Stream Map (VSM) that conducted with the support of the management team together with a group of operators of the hospital. The use of the VSM helped in identifying the wastes and problems in the consultation process and served as a basis to propose improvement strategies.

In addition, a two-ways data and information collection plan has been conducted in order to design, validate, and quantify the built VSM. The following information gathering approaches have been adopted:

1. Qualitative analysis of the process: this was conducted through periodic meetings and interviews with doctors and healthcare staff held by the representative of the management team.
2. Quantitative analysis of the process: data about the consultation times were collected through the hospital information system from January 2018 to December 2018.

### 2.4. Design of the to-be Process: Improvement of the Value Stream and Flow

Once the major causes affecting the increase in the consultation times were identified, the VSM has been redesigned in order to optimize the value stream and flow in order to reduce the process inefficiency and wastes. Among the corrective actions to be introduced, the teleconsulting and the use of handheld portable ultrasound scanners have played a significant role in the re-organization of the process, which took place between January 2019 and December 2020.

### 2.5. Assess and Monitoring the Improvement

A comparison between the as-is and to-be processes has been conducted by means of visual tools (histograms) and statistical analysis (Student’s *t*-test with a 95% confidence interval). Data starting from January 2021 to December 2021 were collected in order to monitor the improvement after the implementation of the corrective actions.

## 3. Results

**Figure 2:**
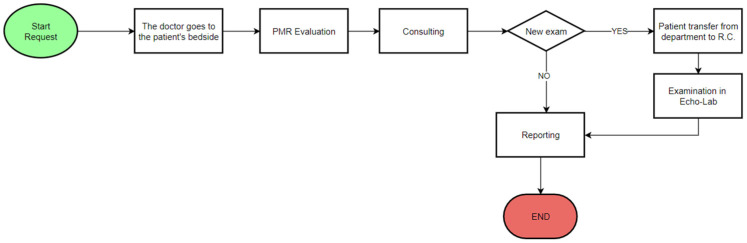
Flow chart of the consultation process before the Lean management intervention.

In the following figure (Figure 2) we report the flow chart before the Lean management intervention.

The process begins with the request for counselling. The specialist proceeds to evaluate the PMR (paper medical records) near the patient’s bedside, examines the patient and decides whether to perform instrumental examinations. If instrumental examinations are necessary, the patient is transferred to the Echo-lab. The process ends after the examination results are reported.

The existing situation was analyzed by observation and VSM; the activation of the Kaizen improvement process took place with the involvement of a multidisciplinary team that analyzed the emerging problems and identified solutions immediately evaluated in the specialist clinic.

Through the mapping of the processes, the following criticalities have been identified and solutions were proposed accordingly, as showed in Table 1.

**Figure 3:**
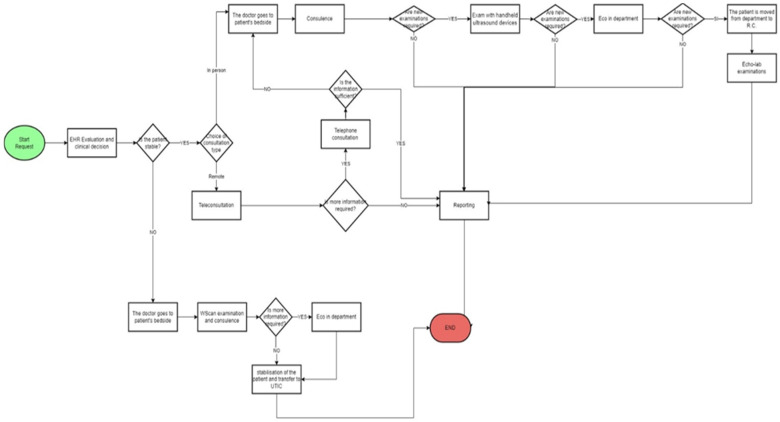
Flow chart of the consultation process after the Lean management intervention.

In the following figure (Figure 3) we report the flow chart after the Lean management intervention with the introduction of the teleconsulting and of the handheld echography instruments.

With the introduction of the Lean method, the specialist doctor does not go directly to the patient’s bedside, but he assesses the patient’s state of health first. If the patient is stable, the doctor evaluates the EHR and chooses how to do the consultation. If the consultation is conducted remotely, and if the specialist doctor considers it appropriate to conduct further investigations, the consultation will be conducted in presence and diagnostic tests can be performed directly at the patient’s bedside. In this case, the new instruments used are the handheld ultrasound devices and the ultrasound machine located in the department where the patient is hospitalized. If these examinations are still not sufficient for a diagnosis, the patient is transferred to the Echo-Lab where the following diagnostic examinations can be performed:

- Physical stress echo
- Pharmacological stress echo

The patient’s condition is critical, the examination is conducted exclusively in presence. In both cases, the process ends after the examination results are reported.

**Figure 4:**
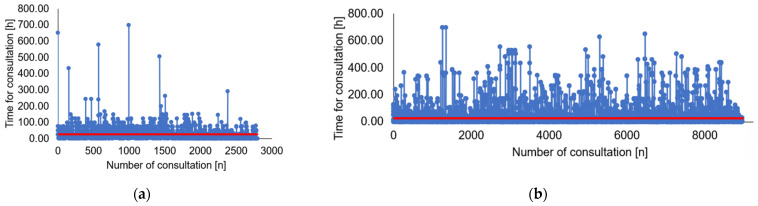
Process flow chart: (**a**) before Lean intervention; (**b**) after Lean intervention. The *X*-axis shows the numbers of consultations. The *Y*-axis shows the time to complete consultation. Average waiting time for consultations is reported in red (solid line).

The reduction of the average waiting time can be observed in the following Figure 4.

The horizontal axis represents the number of medical consultation request. The vertical axis represents the difference between the time of when the consultation is requested and the time of when the consultation is conducted. The comparison between Figure 4a,b shows that the average waiting time for consultation is reduced and the number of consultations provided in a year increase.

**Figure 5:**
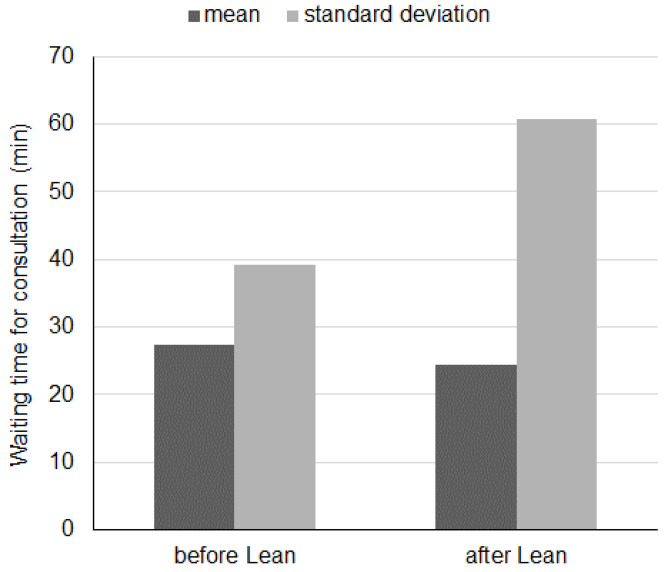
Comparison of average waiting times for consultations before and after improvement.

The reduction of the average waiting time can be observed in the following Figure 5.

Bar graph describing the variation in waiting time for consultation before and after lean approach. The *X*-axis shows the different mean and standard deviation. The *Y*-axis shows the waiting time for consultation ranging from 0 to 70 min.

**Figure 6:**
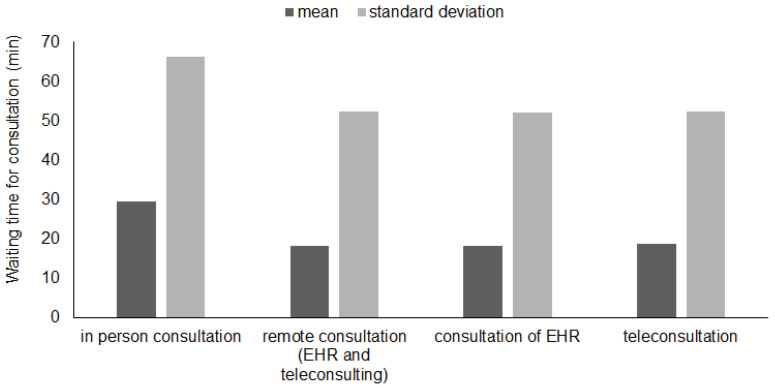
Comparison of average waiting times for each consultation approach before and after improvement.

The higher standard deviation can be explained by the larger variability in the requests due to the COVID-19 pandemic for patients hospitalized in 2021. The reduction of the average waiting time for different consultations approach can be observed in the following Figure 6.

Bar graph shows the result of waiting time for consultation with different approach: in person consultation, remote consultation, consultation of EHR and teleconsultation. Consultation of EHR is any consultation requested by physicians in other specialties done by reviewing only the Electronic Health Record (EHR) without direct patient contact.

Table 2 shows the average waiting times evaluated, before and after the introduction of the improvements, through statistical tests with a significance level of 0.05 using the mean and standard deviation. Considering the average waiting time for total consultations, a percentage reduction of 10.9% is noted. The average waiting time for in person consultation increases by 7.6% unlike other cases where there is a reduction of about 30%. The *p*-values obtained show that these variations are statistically significant.

Table 3, on the other hand, shows the result of statistical comparison between the in-person and remote consultation in the post-improvement phase. 

## 4. Discussion

Consultations with specialists are essential in a process of patient care that can be considered quality. This work provides a Lean intervention to improve the quality of consultations in hospital rehabilitation cardiology.

### 4.1. Previous Studies

From a careful analysis of the literature, the consultations most analyzed are psychological ones or those relating to palliative care [34,35]. The cardiology consultations mainly concern an analysis of comorbidities. In fact, the patients admitted to a non-cardiology ward and in need of a cardiology consultation are the elderly with a high prevalence of heart disease and high hospital mortality [36]. Furthermore, one of the most frequent causes of cardiology consultation is the pre-operative cardiology risk assessment [10,11,12,13]. With a steadily decreasing morbidity and mortality due to non-cardiology procedures, more attention is paid to the management of cardiovascular disorders to guarantee long-term effects. Preoperative cardiology consultation may represent an opportunity to initiate or modify cardiology care, already in primary and secondary preventive measures [37].

Given the massive use of in-house specialist advice, increasing the effectiveness of these interactions is essential for patient-centered care [2]. The problems associated with this process, however, are many. The appropriateness and correctness of the request, the waiting time for delivery and the duration of the visit are just some of the elements to be evaluated [38,39]. Studies have in fact shown that a poor quality of requests and of the subsequent consultation provided cause an increase in the risk of repetition of unnecessary diagnostic procedures, delaying obtaining the best diagnosis and subsequently delaying the start of treatment, increase the risk of medical mis-management, excessive costs of treatment, ultimately negatively affecting patient con-fidence [40,41].

Since the healthcare organizations are based on highly structured and complex processes resulting in high costs and, sometimes, in low quality of services and, consequently, poor levels of customer satisfaction, there is a good opportunity to improve the processes by adopting managerial tools like the Lean Thinking.

The goal is therefore to make the value flow, understood as that for which a cus-tomer/patient would pay, at each stage by reducing the activities that do not add value (waste) which often cause delays, require extra resources, and therefore further costs, and which ultimately lower the quality of the process [42].

**Figure 7:**
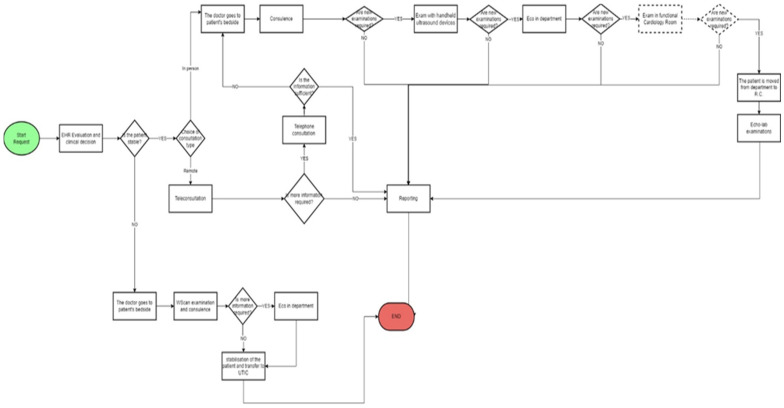
Flow chart of the consultation process after the Lean management intervention with the presence of Functional Cardiology Room.

Although Lean was born in an industrial production context, service organiza-tions soon began to think about an implementation to improve the effectiveness and efficiency of processes. The transfer of Lean to healthcare is relatively new [43], but there is now several evidence of widespread consensus demonstrating the potential of Lean; with significant benefits obtained in the areas of costs, waiting and patient safety [44]. With Telemedicine [45,46] it is possible to further improve secure access to specialist skills through a web system that effectively reduces diagnosis and therapy times [47]. This could lead to the creation in each pavilion of a “Functional Cardiology Room” equipped with a echocardiogram machine and eletrocardiograph machine both connected to the Cardiology Division/Echo—Lab for teleconsulting expert opinion if needed. This would avoid the continuous external movement of the patient who can be examined directly at the bedside through the use of portable devices (FoCUS [48]) and carried to the Functional Cardiology Room of the department only when necessary for a more detailed echocardiogram and/or transoesophageal echocardiography. Thus the patients have to be referred to the central Echo-Lab only for echo stress tests and for particular TTE/TEE exams (Figure 7).

### 4.2. Uniqueness of the Present Study

Our study shows how a combined approach between Lean Thinking and Telemedicine have brought significant benefits to the cardiology consultation process of A.O.R.N. “A. Cardarelli” (Naples, Italy). Comparing the data for year 2018 (pre-intervention) with those for year 2021 (after intervention), it is observed that a reduction in the average waiting time benefits waitlist with a greater number of patients treated in a year. The year 2021 is also the year in which Italy, like other countries in the world, had to face the COVID-19 pandemic. For this reason, there is an increase in the use of remote consultations which makes it possible to further reduce the waiting time compared to that in person of over 38%.

The peculiarity of the study is to apply an innovative methodology such as Lean Thinking in optimizing an important process such as that of cardiology consultations, currently little studied in literature.

### 4.3. Clinical Implications, Limitations, and Future Directions of the Study

The main clinical implication lies in this optimization, with better management of resources and a reduction in waitlist, which produces an increase in the quality perceived by the patient. Our work is not without limitations. First of all, only two years are compared without analyzing any differences in the execution times of the visits due to the complexity of the cases treated or to emergencies.

Future developments, therefore, will involve overcoming the limits set out, also using data mining techniques, already used in various fields of diagnostics [49,50,51,52,53,54] and optimization of health processes [55,56,57,58,59,60,61], for the study of correlations with clinical and demographic variables of patients.

## 5. Conclusions

The objective of the project developed at the A.O.R.N. “A. Cardarelli” of Naples (Naples, Italy) has implemented the lean methodology as a tool to improve cardiology consultation processes. The lean method supported the team members in defining the project and implementing corrective actions to improve the process in question. Statistical analysis was used to characterize the benefits obtained from a Lean approach. The application of corrective actions, with the introduction of portable devices and telemedicine, led to a reduction in the percentage of waiting for counseling from 29.6% to 18.3%. Furthermore, corrective actions have increased the number of consultations provided in a year which went from 2799 (year 2018) to 8979 (year 2021).

Therefore, the benefits of this new process are manifold for both patients and medical staff. For patients, a reduction in the duration of the consultation and an increase in the number of services provided is expected, with consequent satisfaction for the health service. In addition, for the hospital, there is a reduction in costs and an improvement in health quality. In addition, the results of the study show that the introduction of digital tools is useful to improve the level of assistance and optimize performance. The correct allocation of resources, understood as the reduction of waste, is now fundamental, even in health care.

## Figures and Tables

**Table 1:** Causes and proposed solutions to improve the process.

Process Activity	Cause of Inefficiency	Proposed Solution
The doctor goes to patient’s bedside	Waste of time and resource	Teleconsultation
PMR Evaluation	Can be used by one user at time Paper deteriorability Space occupation	EHR Evaluation
Patient transfer from department to cardiac rehabilitation	Elevated risk of infection and worsening of the patient’s health condition	Handheld ultrasound devices and echocardiographic exam in department

**Table 2:** Statistical comparison between the as-is and the to-be processes.

Parameter	Before Intervention (min)	After Intervention (min)	Percentage of Reduction (%)	*p*-Value
Mean waiting time for overall consultations	27.5 ± 39.2	24.5 ± 60.7	−10.9	**0.000**
Mean waiting time for in person consultations	27.5 ± 39.2	29.6 ± 66.4	+7.6	**0.000**
Mean waiting time for remote consultations (EHR and teleconsulting)	27.5 ± 39.2	18.3 ± 52.2	−33.4	**0.008**
Mean waiting time for EHR consultations	27.5 ± 39.2	18.1 ± 52.2	−34.2	**0.024**
Mean waiting time for teleconsultations	27.5 ± 39.2	18.9 ± 52.4	−31.3	**0.006**

**Table 3:** Statistical comparison between the in person and remote consultation after the Lean intervention.

Parameter	In Person Consultations	Remote Consultations	Percentage of Reduction (%)	*p*-Value
Mean waiting time (min)	29.6 ± 66.4	18.3 ± 52.2	−38.2	0.000

## Data Availability

The datasets generated and/or analyzed during the current study are not publicly available for privacy reasons but are available from the corresponding author on reasonable request.
